# Progress in Bacteriology

**Published:** 1903-03-21

**Authors:** 


### Progress in Bacteriology.

**Plague.**—Novy[1] recommends that for purposes of diagnosis a few drops of fluid be drawn off from the buboes, and films and cultures made. If pus be present in the bubo the presence of plague bacilli may be obscured by pyogenic organisms, and it is then best to make cultures from the blood in which the bacilli abound if the case be at all severe. In pneumonic plague the sputum is rich in bacilli, and in post-mortem examination they are easily detected in the glands and spleen. The bipolar staining is well brought out with methylene or carbolthionin blue. If the post-mortem examination has been delayed, rounded involution forms appear. Inoculation of cultures into guinea-pigs is not always followed by death. An important contribution to the morphological and cultural characters of the bacillus pestis is given by Galli-Valerio[2] in an article profusely illustrated by valuable plates. His results may be summarised as follows:—It is better to grow cultures at a temperature of 20° C. than in a warm incubator. Growth in gelatine is visible on the second day, and on agar in from 18 to 24 hours. The colonies are white in the centre, with a clearer peripheral zone, and undulating margin. The most rapid growth is on sloped gelatinised rabbit serum. Lactose is slightly fermented, glucose not at all. Milk is not coagulated. Ziehl's carbol-fuchsin makes an excellent stain, and as the B. pestis is decolorised by Gram, this stain is useful for differential purposes. The organism is polymorphic, coccal, oval, chain, club, and rod-shaped forms being obtained according to the medium used. As a guide to the atypical forms the plates accompanying the article will be found invaluable. In the opinion of the writer the organism does not form a capsule. The organism most closely resembling the B. pestis is the B. pseudo-tuberculosis rodentium which also has the power of exciting lesions closely resembling those of plague in guinea-pigs. The chief points which serve to distinguish the B. pseudo-tuberculosis are:—continuous outline of the colonies; absence of involution forms; power of coagulating milk; non-pathogenicity to certain varieties of rats. Cairns[3] reports on the agglutinating property of the blood serum in cases of plague, and gives details of 24 cases occurring in Glasgow on whom over three hundred observations were made. The technique is identical with that of Widal in testing the blood of cases of enteric fever and the difficulty of obtaining a homogeneous emulsion was overcome in the following manner:—Agar slope cultures were first made and to these sterile ·75 salt solution was added and an emulsion made by rubbing the growth of B. pestis off the agar into the salt solution. Such emulsions will remain homogeneous for days, and are useful either for the microscopical or sedimentation methods. The blood serum may be employed in dilutions of 1 in 10, 1 in 25, or 1 in 50. The agglutination phenomenon does not appear until the close of the first week, increases in intensity up till the sixth or eighth week and then declines, although it may be detected as late as five months from the commencement of the disease. The reaction is best marked in severe attacks and in the lighter cases may never be present. Flexner[4] summarises the present position of anti-plague inoculations as follows: Haffkine has shown by experiment that immunity may be conferred on the lower animals. Inoculation does not afford absolute protection against attacks of plague, but the plague incidence amongst inoculated communities is sensibly lowered. The fatality of attacks is lessened and the death-rate of inocculated populations diminished. Several days elapse before protection is acquired, and once acquired it may exist for several months. It is desirable that the serum should have the maximum of virulence and that it be administered in one dose. With regard to the question as to whether serum obtained from horses immunised against plague has a curative value when once the disease is established the evidence at present does not warrant a conclusion. The experiments of Yersin and Lustig would seem to point to an affirmative answer and it is along these lines that successful treatment must be looked for. Besredka[5] has recently pointed out that a rapid and harmless immunity can be conferred by the injection of anti-plague serum, but this is of very short duration and not to be compared to Haffkine's method, although the latter takes from eight to twelve days to develop and is sometimes the cause of serious local and general trouble. Anti-plague serum, he finds, will preserve its immunising power for a long time if kept in sealed glass tubes.

**Rheumatic Fever.**—Investigations of Beaton and Walker[6] confirm the work of Poynton and Paine with regard to the bacterial origin of rheumatic fever. The organism is a micrococcus and was found not only in cases of rheumatic fever but also in chorea. When injected into animals it gave rise to typical attacks of acute rheumatism. The micrococcus has the following characteristics. It is stained by Gram's and Weigert's methods, and by all ordinary dyes; it is not capsulated, and gives no agglomeration reaction. It grows on all the ordinary media especially well if the reaction be decidedly alkaline. On artifical media generally it assumes a streptococcal form, and the only particular in which it differs from the pyogenic streptococci is in that it does not fulfil Marmorek's test so that it can flourish in a fluid medium in which pyogenic streptococci have grown, but from which they have been filtered. Slight acid formation accompanies its growth, but this is not lactic acid. The cocci may be obtained from the blood or from joint effusions. In rabbits the following lesions result:—Fever, wasting, mono or polyarthritis, pericarditis, endocarditis, septicæmia, and death. Spasmodic movements resembling chorea were once noticed. Suppuration may be excited by this organism either at the seat of inoculation or in the joints. Further details of the organism are given in another article by Beaton.[7] He states that the coccus may be recovered from the urine, and that in cultures it forms no gas, no indol, and no hydrogen sulphide. It is a facultative anærobe. With a culture supplied by Beaton, Shaw was able in rabbits to cause ulcerative endocarditis with multiple infarcts. Several points still demand investigation, more especially the question of specificity both bacterial and toxic. The nature of the acid formed in growth, the isolation of specific toxic products, the pathways of infection, and the discovery of a specific therapeutic agent are all points yet to be determined.

**Growth of Bacteria in the Intestine.**—Lorrain Smith and Tennant[8] find that bacteria are more numerous in the large than in the small intestine. Most varieties ingested with food are destroyed, but proteus and coli survive and flourish. Bacteria only begin to get numerous at the region of the lower ileum and grow most readily beyond the ileo-cæcal valve. The inhibitory power of the stomach is due to the acidity of its contents, but that of the intestine is due to a definite secretion, as is demonstrated by the fact that although the contents of the large bowel are frequently acid, yet it is in this part that bacterial growth is most profuse. The greater the amount of desiccation in the bowels the fewer bacteria in the fæces, and where the desiccation is carried to an extreme, as in the guinea-pig, the contents of the large intestine show the same relatively sterile condition as do the contents of the small. As before mentioned the bactericidal properties are due to the secretion of the intestinal glands, but this is largely aided by the antiseptic power of the bile. Experiments showed that if a portion of the small intestine were congested, or in any way diseased, there immediately resulted a large increase in the number of bacteria in that portion. The experiments were made on rabbits and guinea-pigs, but identical results were secured from human intestine which was examined a few hours after death. The practical bearing of these experiments is obvious. Bacteria ingested by the mouth, such, for instance, as B. typhosus, will first find their opportunity of flourishing in the lower part of the ilium; here the congestion excited by their presence and activity will also permit the increase of other bacteria, such as coli and termo, and so a double infection may occur.

[1] Treatment, April, 1902. [2] Brit. Med. Jour., Sept. 27, 1902. [3] Lancet, June 22, 1901. [4] Univ. of Penna. Med. Bull., Nov., 1902. [5] Ann. de l'Inst. Pasteur, Dec. 25, 1902. [6] Brit. Med. Jour., Jan. 31, 1903. [7] Practitioner, Feb., 1903. [8] Brit. Med. Jour., Dec. 27, 1902.

